# Challenges in shared decision making in advanced cancer care: a qualitative longitudinal observational and interview study

**DOI:** 10.1111/hex.12434

**Published:** 2015-12-16

**Authors:** Linda Brom, Janine C. De Snoo‐Trimp, Bregje D. Onwuteaka‐Philipsen, Guy A. M. Widdershoven, Anne M. Stiggelbout, H. Roeline W. Pasman

**Affiliations:** ^1^ Department of Public and Occupational HealthEMGO Institute for Health and Care Research, Expertise Center for Palliative CareVU University Medical CenterAmsterdamThe Netherlands; ^2^ Department of Medical HumanitiesEMGO Institute for Health and Care ResearchVU University Medical CenterAmsterdamThe Netherlands; ^3^ Department of Medical Decision MakingLeiden University Medical CenterLeidenThe Netherlands

**Keywords:** communication, end of life, patient participation, shared decision making, treatment options

## Abstract

**Background:**

Patients' preferences and expectations should be taken into account in treatment decision making in the last phase of life. Shared decision making (SDM) is regarded as a way to give the patient a central role in decision making. Little is known about how SDM is used in clinical practice in advanced cancer care.

**Objective:**

To examine whether and how the steps of SDM can be recognized in decision making about second‐ and third‐line chemotherapy.

**Methods:**

Fourteen advanced cancer patients were followed over time using face‐to‐face in‐depth interviews and observations of the patients' out‐clinic visits. Interviews and outpatient clinic visits in which treatment options were discussed or decisions made were transcribed verbatim and analysed using open coding.

**Results:**

Patients were satisfied with the decision‐making process, but the steps of SDM were barely seen in daily practice. The creation of awareness about available treatment options by physicians was limited and not discussed in an equal way. Patients' wishes and concerns were not explicitly assessed, which led to different expectations about improved survival from subsequent lines of chemotherapy.

**Conclusion:**

To reach SDM in daily practice, physicians should create awareness of all treatment options, including forgoing treatment, and communicate the risk of benefit and harm. Open and honest communication is needed in which patients' expectations and concerns are discussed. Through this, the difficult process of decision making in the last phase of life can be facilitated and the focus on the best care for the specific patient is strengthened.

## Introduction

Most patients want to have a role in treatment decision making, together with their physician.^1^, ^2^, ^3^, ^4^, ^5^ However, differences are found in the preferred level of participation between patient populations,^6^ for example based on demographic factors such as age,^7^, ^8^, ^9^ and educational level.^9^, ^10^ A review that focused on decision making about palliative care options found that the majority of patients prefer to participate to some degree in treatment decision making, while a substantial minority (13–35%) prefer to delegate the decision‐making role.^11^ From a qualitative study it is known that patients' preferred role also depends on the treatment aim; patients want to be more decisive when their quality of life is more at stake.^12^ Given that patients envisage a more active role further on in the disease trajectory, physicians should be aware of this shift and provide room for patients to be actively involved.

Shared decision making (SDM) may be considered as the ideal model.^13^, ^14^, ^15^, ^16^, ^17^ A central element of common definitions of SDM is the information exchange between physicians and patients and the involvement of both parties.^13^, ^18^, ^19^, ^20^ Stiggelbout *et al*. have distinguished four steps in SDM.^21^ The first involves outlining all options, including the option of doing nothing or keeping the status quo and mentioning that there is no best option, thereby ‘creating awareness of equipoise'. In the second step, the risks and benefits of every option are explained to the patient and their probabilities, to support him or her in the consideration of the options. The third step is helping the patient in the exploration of his or her ‘ideas, concerns and expectations about the options'. The last step involves sharing the responsibility for the decision by establishing an equal partnership and assessing the preferred role of the patient in the decision‐making process.

Yet, little is known about how SDM is used in clinical practice and its effect on patient participation in end‐of‐life decision making. Most studies on SDM have been conducted in the curative setting where patients often have to choose between treatments that have both proven to be effective.^22^, ^23^, ^24^, ^25^, ^26^ In the non‐curative setting, decisions differ substantially from the curative setting, as uncertain gains in terms of survival outcomes and quality of life have to be weighed against the side‐effects of treatment regimens. Second and third lines of chemotherapy in the advanced cancer setting particularly have a limited likelihood of response and only modest improvement in (progression‐free) survival^27^ and are sometimes prescribed to maintain quality of life. Patients' preferences and expectations should be taken into account^11^ in this delicate process of decision making. However, the available data show that SDM is not optimal in non‐curative care. A longitudinal study on terminally ill patients found that these patients did not perceive that their participation in treatment decision making reflected their preferences.^28^ A study in which consultations were tape‐recorded found that only 44% of patients was offered an alternative to anticancer treatment during those consultations, and only 30% were offered a choice.^29^ Koedoot *et al*. also observed that the alternative option of ‘watchful waiting' was mentioned in only half of the consultations about palliative chemotherapy that they observed, while 87% of these patients preferred a strong role in decision making.^30^

The aim of our study is to gain more insight into treatment decision making in patients with advanced cancer, by examining whether and how the four steps of SDM can be observed in clinical decision making about second‐ and third‐line chemotherapy.

## Methods

### Design

In a longitudinal qualitative study we followed advanced cancer patients and their physicians using two methods. Firstly in observations of out‐clinic visits we observed how treatment decision making took place in daily practice. Secondly through face‐to‐face in‐depth interviews after treatment decisions were made we gained insight in patients' and physicians' experiences of and views on treatment decision making.

### Study population and recruitment

Patients diagnosed with either glioblastoma (GBM) or metastatic colorectal cancer (mCRC) were included in the study. These patient populations have a poor prognosis (GBM median survival of 14 months,^31^ mCRC median survival of 24–28 months^32^ and cannot be cured of their disease. When progression of the disease occurs, a decision is often required regarding whether or not to start a (new) chemotherapy aimed at prolonging life, with the potential disadvantage of burdensome side‐effects, such as nausea and fatigue.

Patients diagnosed with GBM or mCRC under the care of the recruiting physicians were eligible for this study if they were aged above 18 years and able to understand and speak the Dutch language. All eligible patients were informed about the study and were handed an information letter by their physician during consultation. Two patients did not receive an information letter because their physician judged that it was too burdensome for these patients to participate in the study. After 1 week, the researcher (LB) phoned the patients and explained the study aims and methods. Patients diagnosed with either GBM or mCRC were included when they had started first‐line treatment to capture future decisions concerning their treatment.

During the inclusion period, 30 GBM patients were approached to participate in this study. 12 declined: four were not interested in the study, five felt they were too ill to participate, one said it was too emotionally demanding because she had problems with her speech, one was too worried about how the disease would develop in the future, and another did not want the researcher to attend patient–physician conversations. Eleven mCRC patients were approached to participate; one refused because of lack of interest.

**Figure 1:**
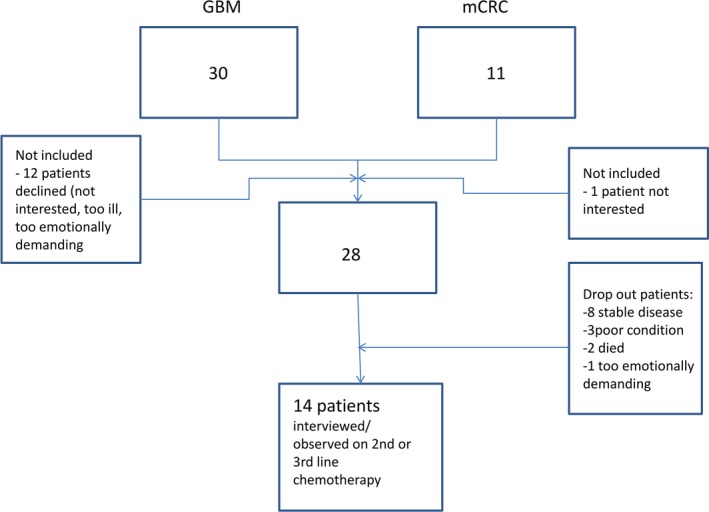
Flowchart of inclusion.

This resulted in 28 participating patients of whom 18 were diagnosed with GBM and 10 mCRC. The patients ranged in age from 27 to 82. Of the 28 patients who were interviewed at the time of inclusion, the status of eight remained stable during the study which meant that no new treatment decisions were made, three dropped out because of their poor clinical performance, and in two, the disease progressed quickly and they died suddenly. One patient dropped out of the study, because involvement was too emotionally demanding when the disease progressed (see Fig. 1).

The remaining 14 patients were confronted with the decision whether or not to start second‐ or third‐line chemotherapy and were part of the analysis within this study (Table 1). Observational data of these 14 patients were used for analysis. Only 13 patients could be interviewed afterwards, because one patient had problems with his speech. In total 18 physicians of different specialties were involved when treatment decisions were made.

**Table 1:** Patient characteristics and number of observations and interviews

Patient (gender, age range)	Diagnosis	Number of outpatient clinic visits observed	Number of outpatient clinic visits in which decision was discussed	Interviews used for analysis		Physicians involved during disease trajectory
				Patient	Physician	
Man, ≤35	GBM	28	7	1	2	1 oncologist
						1 neurologist
						1 neurosurgeon
Man, 51–66	GBM	10	5	2	3	1 oncologist
						2 neurologists
						1 neurosurgeon
Man, 51–66	GBM	2	1	1	2	1 neurologist
						1 oncologist
Man, 36–50	GBM	6	2	1	3	1 neurologist
						1 oncologist
Man, 51–65	GBM	7	1	1	1	1 oncologist
Man, 66–80	GBM	9	4	1	1	1 neurologist
						1 oncologist
Man, 66–80	GBM	11	3	1	–	1 oncologist
						1 neurologist
						1 neurosurgeon
Man, 51–65	GBM	16	6	2	–	1 oncologist
						2 neurologists
						1 radiologist
Man, 36–50	GBM	24	6	3	–	2 oncologist
						2 neurologist
						1 neurosurgeon
Woman, ≤35	GBM	8	1	2	1	1 neurologist
						1 oncologist
Woman, 51–65	Metastatic colorectal cancer	16	2	2	2	1 oncologist
Man, 51–65	Metastatic colorectal cancer	12	4	2	1	1 oncologist
Man, 66–80	Metastatic colorectal cancer	12	3	1	1	1 oncologist
Man, ≤35	Metastatic colorectal cancer	14	3	3	1	1 oncologist
		175	48	23	18	22

### Data collection

The study with GBM patients started in May 2010 and patients were included until December 2012; recruitment for mCRC patients occurred from November 2011 to February 2013. Patients were recruited in a large university hospital within the outpatient clinic of either neuro‐oncology or medical oncology. LB attended each outpatient clinic visit and had informal conversations with patients and relatives in the clinic waiting room. The informal conversations were helpful in building rapport. Doing research in this particular field a relation build on trust is necessary. The outpatient clinic visits were audio‐recorded and observed to capture non‐verbal communication. Both the patient and their treating physician were interviewed separately after treatment decisions were made (whether or not to start a new line of chemotherapy) to establish their perceived role and influence in the decision‐making process. We used an interview topic list based on the objectives of the study. The list contained questions about the decision whether or not to start second‐ or third‐line chemotherapy. Open‐ended questions were asked about their perceived participation in these decisions, the communication with their treating physician and how treatment options were discussed.

### Data coding and analysis

For this paper we selected per patient the visits in which treatment decisions were discussed (or made without discussion). To select these conversations, we first listened to all audio files, since discussions could take place in any visit during the treatment phase. For some patients only one visit was used, for others more than one (Table 1). Audio recordings of the outpatient clinic visits in which treatment options were discussed or treatment decisions were made, and the interviews after treatment decisions were transcribed verbatim. We focused on each of the three types of data (outpatient clinic visits, patient interviews and physician interviews). As our study was explorative, we used open coding as described by Strauss and Corbin.^33^ Our coding scheme was based on the steps of SDM formulated by Stiggelbout et al^34^ (Box 1). We chose the model of Stiggelbout *et al*. because it explicitly describes the steps of SDM. Our aim was to gain insight in de process of decision making and we believed this model was most suitable to describe how decisions were made in clinical practice and how this was related to SDM.

Box 1 **The four steps of shared decision making^19^**

1. Creating awareness of equipoise: explaining to the patient that there is no best choice, that a decision has to be made and that doing nothing or keeping the status quo is also an option.
2. Discuss benefits and harms of each option, as well as their respective probabilities.
3. Patients' ideas, concerns and expectations about the options, their benefits and their harms should be elicited, and the patient should be supported in the process of deliberation.
4. A form of partnership should be built in which patients are encouraged and supported in the process to prevent patients from feeling abandoned and that they have to make decisions on their own. And physicians should invite patients to engage to the maximum extent they desire in making this decision.

In the coding procedure, special attention was paid to parts of the observations which dealt with treatment decisions. The researcher (LB) made field notes on outpatient clinic observations, including non‐verbal communication for better understanding of the data. We did not report explicitly on this non‐verbal communication in the manuscript but it was helpful for the analyses, to better understand the context of the conversation and to better recall the situation.

LB used the summaries of the visits to make a case summary of each patient focusing on what was discussed and what not and whether or not treatment options were discussed according to the steps of Stiggelbout *et al*.^21^ Subsequently LB listened to audiotapes of visits in which treatment options were discussed or where results were considered from blood tests (often used for determining the effects of treatment and deciding whether treatment should be continued or not). In coding the interviews, we focused on text sections in which physicians and patients reported experiences of the decision‐making process related to the SDM steps. Results of the analysis of the observations and the interviews were compared later.

The first five observations and interviews were independently coded by the first and last author to generate a list of codes (e.g. emphasis on treatment, alternatives (not) discussed, medical focus, assuming patient wishes) and compared. Disagreement was resolved by discussion. Codes were also discussed with the other researchers and the group worked towards consensus regarding the interpretation of key themes. Relevant extracts which illustrated the main themes were chosen by this group of researchers. Data analysis started during data collection and was an on‐going process. Data were analysed per outpatient clinic visit and interview, and also longitudinally per patient. A professional translator translated the quotes that we eventually chose to illustrate our results.

### Ethical considerations

The study was approved by the Medical Ethics Committee of the VU University Medical Center, Amsterdam. The participating departments gave their approval for the research to be carried out. Informed consent was obtained from all patients taking part in the study and they were free to withdraw at any time.

## Results

### Steps of SDM in daily practice

Below are the results presented according to the four steps of SDM (Box 1). For each step we first present data from observations of visits in the outpatient clinic to gain insight into the process of treatment decision making in daily practice, followed by views and experiences of patients and/or physicians derived from interviews after treatment decisions were made.

#### 1. Awareness of equipoise

The first step in SDM requires that physicians create awareness of the different available treatment options and that they represent equally good choices (see Box 1). We did not observe visits in which the physician outlined all treatment options, including the option of doing nothing, or mentioned that there was no best option. However, we observed visits in which physicians said that when progression would occur, patients could either have chemotherapy or they ‘could do nothing', without further explanation of these options. In some cases starting a second‐ or third‐line of treatment was immediately offered after giving the bad news without offering alternatives. For example, a mCRC patient who had just heard that the disease progressed was offered only the option of treatment:

Patient (P) What are we going to do?

Doctor (D) I really want to start a new treatment.

[…]

P When?

D Today, actually.

P Oh, today. So soon?

D Given your problems and how the metastases have grown in the scan, I don't think we can spend another month waiting to see what happens.

P OK, well I suppose I'm here anyway.

*Observation in the outpatient clinic, mCRC patient, man, aged ≤35, progressive during second‐line chemotherapy*

Another example, is a GBM patient in whom progressive disease was determined on a MRI scan. He was offered second‐line treatment (surgery and chemotherapy) while not starting second‐line treatment was not discussed with this patient:

D We can see new tumour growth after all in the MRI.

P Oh, that's bad news. [The patient is visibly upset].

D A tumour has appeared in a completely different place, the tumour's about 2 × 3 cm. I'd like to discuss it with the team, see what could be done. I think we could operate again with follow‐up treatment. That will be chemotherapy. You've had the standard treatment. The tumour will come back eventually. We'll have to see then what we can do. I think we'll still be able to operate.

*Observation in outpatient clinic, GBM patient, man, aged 51–65, progression after 8 months of stable disease after finishing first‐line chemotherapy*

There were physicians that intentionally did not create awareness of equal treatment options. In the interviews they said that they preferred treatment over no treatment as they believed patients could benefit from chemotherapy although chances were modest. For example, an oncologist said during the interview that he offered third‐line treatment because progression was established and he followed treatment protocols:

Interviewer (I) Was it immediately clear to you that you should start giving third‐line treatment?

D Yes.

I Why?

D Well, because his disease was progressive with liver test values in his blood that had increased sharply, and the CT scan where you saw an increase in liver metastases and lung metastases too. And when that's the case, you just want to carry on giving therapy. That is actually the standard therapy, the therapy I'm giving now.

*Interview with oncologist, talking about starting third‐line treatment of an mCRC patient aged ≤35*

This emphasis on treatment seemed to be related to physicians wanting to maintain hope:

(D) Look, you try to soften the blow a bit by following up the bad news with some hope by offering a new treatment. So in that respect it actually makes the talk easier.

*Interview oncologist, talking about decisions to start a subsequent line of chemotherapy in mCRC patients*

#### 2. Discussing benefits and harms of options and their probabilities

After explanation of the options, the next step of SDM is to discuss the benefits and harms of each as well as their respective probabilities (see Box 1). During the outpatient clinic visits potential benefits and side‐effects of starting a new chemotherapy were often discussed. However, since the option of not continuing treatment was not discussed in the first place, benefits, harms and probabilities of this alternative were hardly ever addressed in these visits.

When benefits, harms and probabilities of starting a new chemotherapy were discussed, this was generally not very extensive. From the interviews with patients it seems that this is related to many patients being focused on continuing treatment, as is shown in the following example of a patient who was interviewed after the decision was made to do another brain surgery:

I If you look at what a treatment like that involves for you and all the side‐effects, how does that weigh up against the benefit you get from it?

R Um, I haven't really thought much about that. In part because at the moment at any rate I'm in a kind of state where I go for everything they offer and every option so that, well… to let things stay OK for as long as possible.

[…]

I But did they tell you well… what you gain from this treatment?

R No, they didn't mention that.

*Interview, GBM patient, man, 36–50 years, decision made to start second‐line chemotherapy*

This focus on continuing treatment seemed, at least for some patients, related to fearing they might experience regret if they decided not to start this new treatment:

I But you… let I put it this way, you are motivated enough to start with it? [second line chemotherapy]

P Yes. Well, partly because I think that if you do nothing… well, I always think, what if you'd done nothing and it had returned, then you'd always wonder whether perhaps you should have done something.

*Interview, GBM patient, man, aged 36–50, decision was made to start second‐line chemotherapy*

In three patients it was decided not to start a subsequent line of chemotherapy. Although these patients received similar information as the other patients, they stated afterwards that the low chances of the potential benefit of the chemotherapy to stabilize tumour growth for them did not outweigh negative effects on quality of life. For example, a GBM patient said after the decision not to start chemotherapy in the interview:

P I went over there [outpatient clinic] with the idea that it was probably not good. And we immediately received that message. And yes, there were options for something like second‐line chemotherapy but those chances were so small, not really what we… they were pretty depressing. Then we were given time to think it over. […] and when you then get something like a 15% chance that it might… that the tumour stops or is held back… that's really not much.

*Interview, GBM patient, man, aged 36–50, decision made not to start second‐line chemotherapy*

These patients based their decision on the information they got about the benefits and harms of *starting* chemotherapy. They did not get information about the benefits and harms of stopping chemotherapy.

#### 3. Patients' concerns and expectations

The third step of SDM involves eliciting patients' ideas, concerns and expectations about the treatment options and their benefits and harms and supporting the patient in the deliberation process (see Box 1). We observed that patients' concerns and expectations were only partly discussed in daily practice and in some patients not asked for at all. We saw that some patients experienced side‐effects or limitations caused by the treatment regimen that influenced their quality of life. For example, a patient diagnosed with GBM with comorbidity experienced discomfort due to his catheter (needed because of problems with his prostate). Although he mentioned this during the consultation, the physician did not regard this as a priority for the patient and said this problem could be postponed until after the chemotherapy for the brain tumour wasfinished:

P And round about then there's the prostate that we'll need to…

D We'd decided to put that off until after the cycles. After the cycles you'll have another 4 weeks until the effect has worn off. Then we'll have the scan four to 6 weeks later and then I'll ‘release' you, as it were, and the urologist can get to work. The problem will be manageable if we do that, won't it?

P I'm not in pain, it's more that I have problems with the leg bag, it's leaking and things.

D I hope that the urologist will be able to do something about that too, but after the cycles.

*Observation in outpatient clinic, GBM patient, man, aged 66–80, 5th cycle of chemotherapy in first‐line chemotherapy*

We also observed that physicians mainly focused on the physical condition of patients, such as in the following example where a patient tries to bring up how he experienced the bad news:

D How's things?

P Well… How should I put it? I've obviously had bad news.

D [interrupts the patient] How is it going in neurological terms? Well, you'd already had that bad news, hadn't you? Last week from Dr …. And then we started with dexamethasone. That's what I'm really interested in, how it went after you started that.

*Observation outpatient clinic, GBM patient, man, between 36 and 50 years old, progression after finishing second‐line chemotherapy*

These quotes illustrate the different views for patient and physician of what is important for them. While the patient is dealing with the bad news he received, the physician focused on his physical condition. However, for SDM the patient's expression of concerns should be a starting point to talk about ideas, concerns and expectations. Instead the physician mainly focused on the physical condition of the patient while an exploration of the patient's ideas and needs would be more appropriate and helpful to support the patient in the deliberation process.

Ideas and expectations about survival gain of a second or third chemotherapy were not always similar between patients and physicians and not discussed explicitly during visits. Some patients had unrealistically high expectations of their treatment in terms of survival. For example, a patient with mCRC said during an interview after starting a third‐line treatment that she hoped to continue with this treatment for half a year, while the physician said during the interview that most patients have progressive disease after two cycles, which meant 6 weeks in practice:

I Do you think the physician wouldn't have offered it [third line treatment] if she expected it wouldn't work out well for you? Do you think the physician would only offer you the treatment if there is a chance of effect or do you think the physician will mention all the available options?

P Well, I reckon this was the best option for me at that point, I assume that's the case if they offer that, yes. Look, the way I see it: imagine I can cope with this again for 6 months, I can do that… just like with the chemo [first‐line and second‐line], they also both did their job for 6 months, perhaps they'll have found something else in those 6 months that might give me a better chance. Look, I'm going to die, right? This cancer will take over. But if something can put that moment off, I'll be prepared to try it, won't I? And just as I am now, so that I've still got some quality of life, as it were.

*Interview, woman mCRC, aged 51–65, decision to start third‐line treatment*

Another example was of a patient in whom progression of the disease was established through a CT scan. When the results were discussed the conversation focused on treatment details, such as length and side‐effects. The benefits in terms of survival and effects on quality of life were not brought up by the physician or by the patient.

During an interview a physician said that he knew what the patient thought about whether or not starting a new chemotherapy in case of progression of the disease and that there was no need for explicitly asking this when progression would occur:

I If I remember correctly, you started the conversation with an advice for a new therapy. On one hand it provides hope, but to what extent did it influence the outcome [starting third line treatment] of the conversation?

D I don't get the impression that he wants to stop.

I No. Is that what you feel?

D Yes, but I can also tell it from what he says to me. He's afraid it won't work anymore and what will we do if it no longer works: is there anything else? So he wants to continue, he hasn't yet reached the point of calling it a day. And then, if you ask what should we do – should we stop or should we continue – well, there's only one possible answer, isn't there?.

*Interview with oncologist, talking about the decision to start third‐line treatment in an mCRC patient aged ≤35*

Some patients experienced that the focus was mainly on their disease or physical condition. For example, during an interview a GBM patient said that the focus was mainly on his disease during his outpatient clinic visits, i.e. whether it was stable or had progressed and not on his functioning in daily life or on considerations whether or not to start new treatment:

I As you explain it now, with all those things you take into consideration about why you should or shouldn't do it: do you feel you can discuss that with…?

P Well, perhaps I… I don't know… I'm not afraid to discuss it, but somehow I don't have the feeling that it's very… easy to discuss that kind of thing out in the open.

I No, but should there be more room for that? Do you feel a need for this?

P Yes, I think so. More room for what considerations there are exactly, what are the pros and cons and how you see things, really like we are discussing it. Yes, I think that could also be part of the talk with a doctor. Of course they would bring in their own expertise in examinations and the options. So that can be of added value.

*Interview, GBM patient, man, aged 36–50, progression after finishing second‐line chemotherapy*

#### 4. Shared process, partnership and desire to participate

In SDM, a form of partnership should be built in which patients are encouraged and supported in the process to prevent patients from feeling abandoned and having to make decisions on their own. Physicians also should invite patients to engage to the maximum extent they desire in making decisions (see Box 1). We observed that some physicians tried to involve patients actively when decisions concerning their treatment were made, but this was after they had already expressed their own preferences. We observed that physicians mostly suggested or advised to start a new treatment. This is illustrated by a visit of a GBM patient to his neurosurgeon in which a second surgery was discussed because of tumour progression. The neurosurgeon tried to involve the patient in the decision‐making process, but steered towards treatment, by offering two options, both involving chemotherapy.

D What can you expect from this? We can't remove everything. So it'll only be temporary anyway. The first time, we had quite a long interval without the disease. We know that a second cycle of treatments can be quite a bit less effective. Although we don't actually even know how big or small that effect is. We won't be able to keep things under control for 2.5 years again. I'm thinking in terms of an extension of months with the operation. An extension of months with the operation, rather than 2.5 years. We don't know whether it'll be 2 months or ten, but in practice it's likely to be somewhere in the middle. So that's really the question for you: are you prepared to run the risk of surgery with an unknown benefit with the aim of keeping it under control again for a while?

[…]

D Now we've got two options: chemotherapy without surgery or chemotherapy with surgery. It's also up to you, you just said you were in favour. I am too, so I reckon that's what we're ending up with.

*Observation in outpatient clinic, GBM patient, man, aged ≤35, progression after a period of stable disease after finishing first‐line treatment*

Furthermore, in outpatient clinic visits we observed that patients were sometimes invited to make up their mind. For example, a patient with mCRC had to decide if he wanted to start a combination of intravenous chemotherapy and oral chemotherapy, because only oral chemotherapy was not sufficient:

D There will come a point when things start to get worse. The chemotherapy may shift that point. It may come sooner if you don't have treatment. But the treatment does have side‐effects. These are temporary negative effects and you will need to be prepared to cope with them, or not, with the aim of possibly extending your life. And if you say I don't think the benefits outweigh the downsides for me, that's something you really will need to take a decision about at a certain point. At the moment, we're saying that we'll see how it goes because you're feeling fine. But there will come a time when things start to get worse and then you'll need to take a decision.

P [The patient, talking at the same time] Yes, of course.

D Whether you want to try to extend it or not. And you will need to get a clear idea about this.

*Observation in outpatient clinic, man with mCRC, aged 66–80, decision whether or not to restart first‐line chemotherapy*

Although this physician supported the patient and explained harms and benefits of both options, he put the decision in the patients' hands.

Some physicians perceived a strong wish in patients to try another treatment. They were willing to prescribe chemotherapy if such patients were in a relatively good clinical condition. In these situations physicians perceived an active role from their patients in the decision whether or not to start a new treatment.

I And who took the decision to start the second‐line treatment?

D Well, he did, I reckon.

I Yes, but you thought… you agreed with him?

D Oh yes. Well, personally, if it had been my father I wouldn't have recommended it. But from a clinical or physical point of view, let's say, his condition was good enough. He'd never had problems with his blood. So yes, in that sense there wasn't really any medical reason for saying he couldn't do it.

*Interview with oncologist, talking about decision to start second‐line chemotherapy in a GBM patient aged 51–66*

Another physician said during an interview that he could only provide adequate information and not decide for his patient, because it is the patient that has to live with the consequences.

I To what extent did you have the feeling to be decisive or make the decision?

D Well, with him I really feel every time that I more or less have to take the decision. And that is actually precisely what I don't want to do. Look, I can say you should do this or you should do that, but – well ‐ the thing is that I know when I do that he doesn't entirely agree, because he's the one who has to take the decision in the end because he will be suffering the side‐effects. So I do try to shift the decision a bit towards him every time so that he really does have to make up his mind. And I think he finds that difficult.

[…]

D Well, I think he'd be happier if you gave him more of a helping hand.

I OK. But you don't think you should be doing this?

D No.

*Interview, oncologist, talking about mCRC patient aged 66–80 where a decision needs to be made whether or not to start intravenous chemotherapy (first‐line treatment)*

However, many physicians saw a decisive role in the decision‐making process from themselves. They said that not all patients were capable of making decisions on their own and they would take the lead in the patients' best interest.

From the patients' interviews we saw that all patients felt they had been involved in the decision whether or not to start a second or third‐line treatment. Compared to their preferred level of participation (before treatment decisions were made), patients perceived they were equally involved or more active when treatment decisions were made. Almost all patients said that the treatment advice of the physician was in line with their wishes and they saw themselves as having the final say. From the three patients for whom it was decided to stop further treatment, two said it was also the advice of their physician and one said he made the final decision.

Some patients, however, felt they were not involved much in the considerations that were made concerning treatment decisions and wanted more information, for example:

I How do you look back on it, about how it went, the conversations with the physician and the infor‐mation you received. Are you satisfied about it?

P Well, I'd like to know a bit more about the considerations when they say to me, for instance, I don't know how many times (number of cycles of chemo). […] They don't really involve you much in the considerations. Especially if there are no real guidelines about what the alternatives should be. And that may well be because it's simply been said that there are no other … that we don't see any alternatives. But then I'd like to know that.

*Interview, GBM patient, man, aged 36–50, decision to start second‐line chemotherapy*

Although this patient understood that there were uncertainties, he wanted to be informed about the deliberation that took place between physicians or in multidisciplinary team meetings.

Overall, patients were satisfied with the decision‐making process; if they were disappointed this concerned the course of the disease. For example, if progression occurred soon after it had been decided to continue with treatment, they were disappointed and felt powerless. Nonetheless, none of these patients regretted that they had chosen to start the treatment. In cases where the patient had stable disease after receiving treatment they were also satisfied, because they believed they had made the right choice.

## Discussion

This qualitative study aimed to gain insight into treatment decision making in the last phase of life of advanced cancer patients by examining whether the steps of SDM are applied in clinical practice. Although some physicians actively tried to invite patients to participate in the process and most patients felt they had been adequately involved, the steps of SDM were hardly ever followed. Patients' awareness of available treatment and care options was limited. In practice, physicians often steered decisions towards treatment without explaining alternatives, because they wanted to maintain hope or follow treatment protocols. Patients felt that starting a new line of chemotherapy was the only option and they feared that they might experience regret afterwards if they did not start chemotherapy. Furthermore, physicians paid little attention to patients' functioning in daily life and quality of life of the patient during the outpatient clinic visits. Patients' concerns and expectations were not explicitly assessed, which led to different expectations on survival gain of subsequent lines of chemotherapy between physicians and patients.

### Strengths and limitations

The main strength of the study is the combination of interviews and observations, which provides an in‐depth and broad understanding of the daily clinical practice and the perspectives of physicians and patients towards SDM in advanced cancer care. Furthermore, the use of steps of SDM clearly shows what barriers and problems apply to SDM in this setting. The fact that the observations and interviews were carried out by one researcher could be considered as a limitation of the study, although potential researcher bias was prevented by frequent meetings with the research team (peer debriefing) during the period of data collection and analysis. This multidisciplinary research team furthermore helped the researcher to be reflexive, which can be seen as a strength of the study. The small sample of patients for whom a decision was made may also be seen as a limitation. However, due to the nature of the sample and the difficulties with recruitment in this setting it was difficult to avoid. Moreover, we found similar patterns in all included patients, strengthening our impression that data saturation had been reached.

Another limitation is the single setting, the study was carried out in one single university hospital and the findings may not applicable in other clinical settings. Finally, it has to be realized that with a qualitative design it is possible to get insight in and understanding patterns that exist in practice, but that it is not possible to show how often these patterns occur.

### Focus on treatment

The focus on treatment options and the lack of addressing the alternative of non‐treatment has been reported previously.^35^, ^36^, ^37^, ^38^ More than 10 years ago, De Haes & Koedoot^38^ already described the preference of oncologists for chemotherapy without good evidence for life prolongation. They recommended that in the setting of palliative cancer treatment, the information should concentrate on the potential effects on quality of life. Although there has been both an increased attention for SDM and substantial development of palliative care in the last decade,^11^, ^37^ it seems that these recommendations have not yet been adequately implemented and that little has changed in the last decade. Our study shows that physicians still emphasize the medical aspects and pay less attention to daily functioning and quality of life.

Furthermore, physicians did not seem to deliberate with their patients regarding the best course of action. They tried to assess implicitly what would be the wish of the patient and the best thing to do. The lack of active patient involvement in the decision‐making process does not seem to point at a negative attitude towards SDM by these physicians, as they are convinced that they do take the patients' wishes into account.

It cannot be concluded that the focus on treatment is due to the physicians, since this study shows that patients also generally opted for treatment. The preference of patients may be related to the fact that they already had experienced the effects of first‐line treatment and therefore expect similar results from the subsequent line of treatment.^35^ The preference for treatment by patients might also be explained by the way the options are presented. Patients may not have been aware or informed of other options. If an alternative was mentioned, the option of not going for second‐line chemotherapy was often presented as ‘doing nothing', implying an end to the involvement of the treating physician and referral back to the GP. But the option of no treatment might become a more equal and applicable option when defined as palliative care, including psychosocial support and pain management.^39^, ^40^ Previous studies show that when recognized that there are no right or wrong decisions in situations of equipoise, just ‘the right decisions for me' based on personal values, this could facilitate patient involvement.^41^, ^42^, ^43^, ^44^

### Mentioning benefits and harms

Informing the patient about the benefits and harms of the treatment option and the alternatives needs further consideration. Probabilities were mostly mentioned in a general sense and in some cases not discussed at all. From our previous study, we know that patients set limits for themselves to preserve their quality of life^12^ and that they would decide to stop their initial treatment if that limit were reached. Furthermore, physicians often put more emphasis on active treatment options than on forgoing treatment. In most cases it was decided to start a subsequent line of chemotherapy, we cannot relate this finding to the information that was given to patients. Yet, it seems relevant how information is given, and the physician's emphasis on particular options may play a role.

In the discussion of benefits and harms, the option of no treatment should be presented as palliative care. Palliative care is focused on improving quality of life in the last phase of life by relieving pain and discussing personal and future wishes for care at the last phase of life.^39^ It has been shown to improve quality of life and even prolong survival, compared to standard (and more aggressive) care, especially when offered early in the treatment process.^40^, ^45^, ^46^

### Hope and anticipated regret

This study showed that in offering palliative care, one should take into account that continuing or starting second or third lines of treatment can be motivated by the intention to foster hope. This has been indicated in previous research, for both physician and patient.^35^, ^38^, ^47^ Buiting *et al*.^35^ called it the ‘facilitating role of chemotherapy' and showed that patients used treatment as a coping strategy to deal with their approaching death. Physicians may feel that taking away the hope of patients would harm patients' wellbeing^47^ and starting a new line of treatment can be seen as a way to deal actively with the cancer.^36^, ^38^, ^48^ Palliative care could prevent patients from getting into treatment only because of a hope for benefit, by giving honest information about prognosis and providing emotional support to get through the hard time in the last phase of life. Even if patients are referred back to their general practitioner, staying in contact with their neurologist or oncologist might give them peace and a feeling of not being abandoned.

The current study shows that patients feared regretting their decision later on and therefore wanted to start a new line of treatment. This anticipated regret has been reported before^49^ and might explain the finding that all patients were satisfied with the way, and extent, in which they had been involved in the decision‐making process. This could represent a kind of cognitive justification: patients were satisfied with the process because they did not want to feel regret about the process or the decision made. They would rather feel good about whatever decision was made, even when the disease progressed or the treatment did not improve their situation.

### Challenges for SDM

According to Charles *et al*.,^13^ SDM is especially important in cancer care. This is because the decisions have a large impact on the health of the patient and the consequences are mainly uncertain, therefore ‘there often is no clear‐cut right or wrong answer'. The answer for each individual can be found within the deliberation between physician and patient to explore what treatment option would best fit for this patient in this situation. In the deliberation, the physician has the role of a ‘teacher or friend' by carefully involving the patient in a dialogue about what values are in play and what health‐related values should be best to strive for.^50^ However, this study shows that deliberation between physician and patient remains a challenge. This requires adequate implementation of the steps of creating awareness of equipoise and mentioning benefits and harms, because only then the values of the patient can be adjusted to the different treatment (and palliative care) options. In addition, from previous research it is known that patients prefer a more active role further on in the disease trajectory.^51^, ^52^ They describe limits for themselves and want to be more decisive if their quality of life is at stake. This corresponds with step 3 of the SDM model in which expectations and values are discussed and step 4 in which the physician should invite patients to engage to the maximum extent they desire to participate. It is also found before that patients foresee a shift in preferences further on in the disease trajectory,^52^ which also aligns with step 4. If steps of SDM would adequately be followed, whishes and expectations of patients would be discussed (step 3) as well as preferred engagement in the decision making (step 4).

Finally, the physician should be encouraged to invite the patient to discuss the values underlying their preferences for treatment, to break the taboo to talk about the approaching death and to take away unrealistic hopes for life cure. By this, the difficult process of decision making in the last phase of life can be facilitated and the focus on the best care for the specific patient strengthened.

## Conclusion

The steps of SDM were hardly ever met in daily practice. To reach SDM in daily practice, physicians should create awareness of all treatment options, including forgoing treatment with chemotherapy, and communicate the risk of benefit and harm. Open and honest communication is needed in which patients' expectations and concerns are discussed. Through this, the difficult process of decision making in the last phase of life can be facilitated and the focus on the best care for the specific patient is strengthened. Further research around integrating SDM into daily practice needs to address the issues found in this study such as preservation of hope and presenting palliative care without chemotherapy or surgery as a good and equal treatment option, not as doing nothing or no treatment. A next step would be to first implement SDM in daily practice and subsequently evaluate this. This could be performed by implementing SDM through for instance physician training and subsequently evaluate the effects of the training on daily decision making and experiences of patients and physicians.

## Source of funding

This study was funded by the Innovational Research Incentives Scheme VICI 2008 from the Netherlands Organisation for Scientific Research (NWO).

## Conflict of interest

None declared.
